# ATP half‐sites in RadA and RAD51 recombinases bind nucleotides

**DOI:** 10.1002/2211-5463.12052

**Published:** 2016-04-06

**Authors:** May E. Marsh, Duncan E. Scott, Matthias T. Ehebauer, Chris Abell, Tom L. Blundell, Marko Hyvönen

**Affiliations:** ^1^ Department of BiochemistryUniversity of CambridgeUK; ^2^ Department of ChemistryUniversity of CambridgeUK; ^3^ Present address: Paul Scherrer InstitutVillingenSwitzerland; ^4^ Present address: Target Discovery InstituteNuffield Department of MedicineUniversity of OxfordUK

**Keywords:** ATPase, homologous recombination, nucleotide‐binding site, RAD51, RadA, specificity

## Abstract

Homologous recombination is essential for repair of DNA double‐strand breaks. Central to this process is a family of recombinases, including archeal RadA and human RAD51, which form nucleoprotein filaments on damaged single‐stranded DNA ends and facilitate their ATP‐dependent repair. ATP binding and hydrolysis are dependent on the formation of a nucleoprotein filament comprising RadA/RAD51 and single‐stranded DNA, with ATP bound between adjacent protomers. We demonstrate that truncated, monomeric *Pyrococcus furiosus* RadA and monomerised human RAD51 retain the ability to bind ATP and other nucleotides with high affinity. We present crystal structures of both apo and nucleotide‐bound forms of monomeric RadA. These structures reveal that while phosphate groups are tightly bound, RadA presents a shallow, poorly defined binding surface for the nitrogenous bases of nucleotides. We suggest that RadA monomers would be constitutively bound to nucleotides in the cell and that the bound nucleotide might play a structural role in filament assembly.

**Abbreviations**
HumRadA2: humanised RadA ATPase domain
RadA‐ct: monomeric ATPase domain of RadA
ssDNA: single‐stranded DNA

Homologous recombination, the process of error‐free repair of DNA double‐strand breaks and stalled replication, has been well conserved through evolution from bacteria to humans 1, 2, 3, 4. This process is facilitated by a group of recombinases that include proteins from the bacterial RecA family 5, the archaeal RadA family 6 and the eukaryotic RAD51 family 7. Double‐strand break repair involves several steps. First, the DNA is unwound and digested to generate single‐stranded DNA (ssDNA) 8, 9. In the presence of ATP and Mg^2+^, the recombinase then coats the ssDNA, forming a nucleoprotein filament. This structure invades the sister chromatid, searching for the homologous stretch of dsDNA, which is then used as a template for recombination‐mediated repair 10.

RAD51 and RadA are structurally very similar, with 46% sequence identity and both containing a C‐terminal ATPase domain and a smaller N‐terminal domain that is implicated in DNA binding 11, 12. The highly conserved ATPase domain contains nucleotide‐binding Walker A and Walker B motifs, in addition to two loops (L1 and L2) that are located close to the nucleoprotein filament axis and are predicted to bind ssDNA 13, 14, 15. RecA contains a similar catalytic ATPase domain as RAD51 and RadA, and performs a similar biological function 7, 14, 16. However, there are differences between the families regarding ATP hydrolysis, co‐operative DNA binding and the polarity of strand exchange 14, 17, 18, 19.

Structural studies have shown that RAD51 and RadA exist in both oligomeric ring and helical filament forms 14, 15, 16, 20, 21, 22, 23. In both forms, oligomerisation occurs via a protein–protein interaction between a short flexible ‘FxxA’ oligomerisation motif (that links the N‐ and C‐terminal domains of one recombinase protomer, Fig. 1A), and the C‐terminal ATPase domain of the neighbouring protomer. The flexibility of this linker region allows a significant degree of conformational freedom between the recombinase subunits. This structural elasticity allows the formation of both ring structures and helical nucleoprotein filament structures of variable pitch. Indeed, electron microscopy and crystallographic studies show that different forms of the helical nucleoprotein filament reflect the state of recombinase activity [13, 14, 15, 20, 22, 24, 25, 26, 27]. In the presence of ATP or nonhydrolysable ATP analogues, an extended ‘active’ form of filament is seen with a helical pitch between 90 and 130 Å. In contrast in the presence of ADP or in the absence of a nucleotide cofactor, a more compressed ‘inactive’ filament form is observed with a pitch of 60–80 Å. Although the mechanism of interconversion between these two forms is unknown, it is likely that nucleotide binding and/or hydrolysis play a crucial role in determining the conformation and thus the activity of the nucleoprotein filament 28.

**Figure 1:**
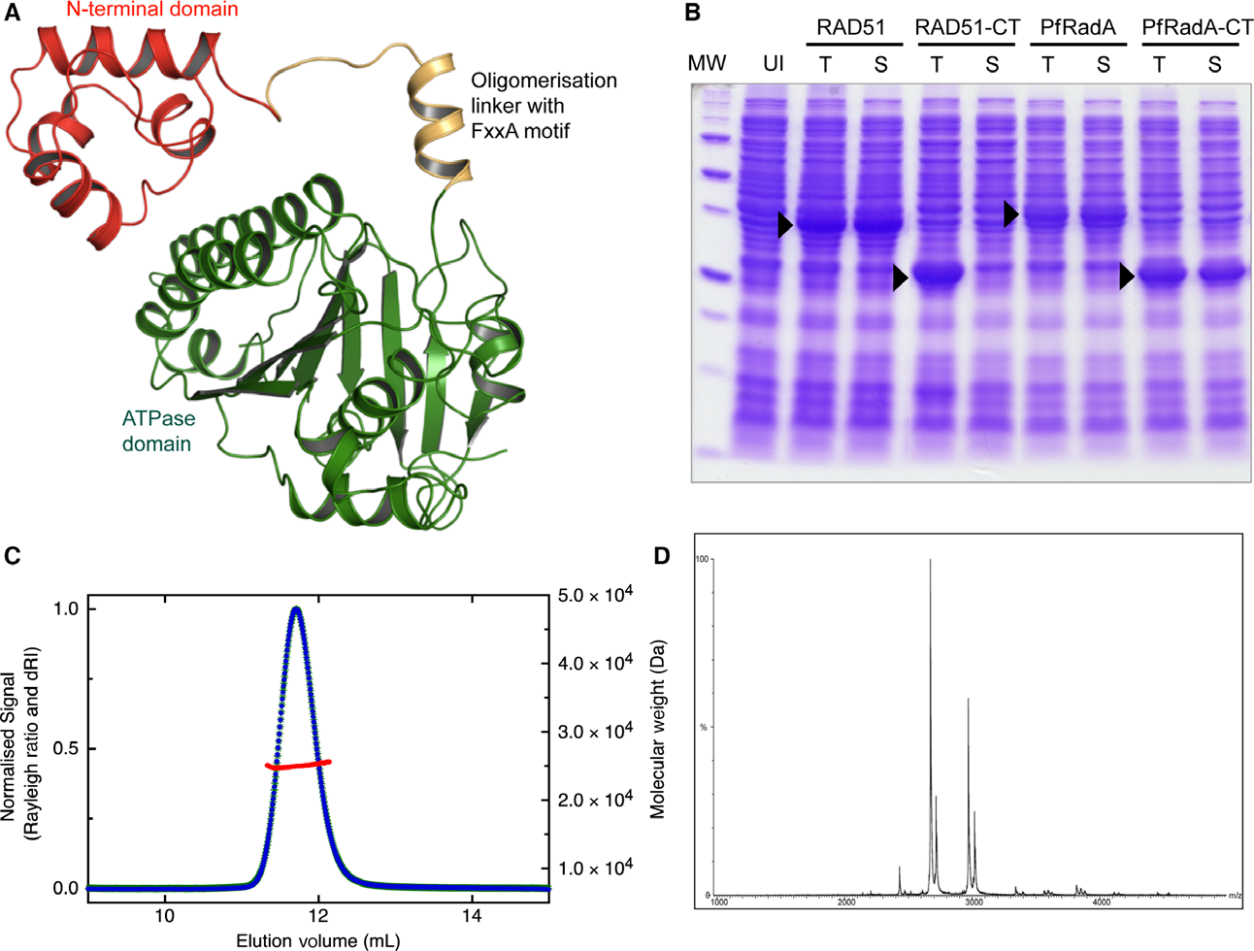
C‐terminal ATPase domain of *Pyrococcus furiosus* RadA is monomeric (A) Domain structure of a single protomer of full‐length RadA from *Pyrococcus furiosus* (PDB: 1PZN) showing the different structural units in different colours (B) SDS/PAGE gel of *E. coli* expression tests for both full‐length proteins and C‐terminal domains of *P. furiosus* RadA and human RAD51; total lysate (T), soluble fraction (S) and uninduced control (UI). (C) Multi‐angle laser light scattering chromatogram showing protein elution monitored by the change in refractive index (blue) and molecular weight calculated from light scattering data (red). (D) Nondissociative mass spectrum showing ions with mass/charge ratios consistent with a monomeric C‐terminal RadA.

Recently, crystal structures of RecA in complex with single‐ or double‐stranded DNA and a nonhydrolysable ATP analogue were determined 20. This filament structure has an extended form with a pitch of 94 Å. A key difference between this structure and the lower pitched inactive RecA filament form is the relative positioning of the ATPase domains. However in the active filaments, the nucleotide‐binding site is buried, and in the compressed inactive form, the site is more open and solvent accessible. These observations are entirely consistent with previous electron microscopy and biochemical data for RecA 29, 30, 31, 32. Although no crystal structures have yet been solved for either RAD51 or RadA in complex with DNA, both have been crystallised in the extended active form 33, 34, 35. These structures show a similar arrangement of ATPase domains as seen with the DNA‐bound RecA structure. Differences in the conformations of the L1 and L2 DNA‐binding loops are observed between the crystal structures of active and inactive filament states across all members of the recombinase family. In the inactive filament state, the loops are either completely disordered or present in multiple conformations 20, 24, 25, 36, 37; in contrast, these loops are more ordered in the active filament forms 20, 34, 35, 38.

At the intersubunit interface of the extended filament structure of *Saccharomyces cerevisiae* RAD51, a direct interaction is observed between the nucleotide‐binding pocket and histidine 352 (His294 in human RAD51, His307 in *P. furiosus* RadA) of the adjacent protomer 33. This residue is highly conserved among RAD51 and RadA recombinases. The additional intersubunit contact that it provides is observed only in the active filament form, possibly providing additional stability to the active filament conformation. The equivalent residue in *Escherichia coli* RecA, Phe217, has been shown to have a regulatory role in the transmission of allosteric information across the subunit interface of the RecA filament 29. The *S. cerevisiae* RAD51 filament structure was found to have two types of protomer–protomer interactions at the ATPase site with the His352 residue taking a different conformation in each. In one interface, it points towards the nucleotide‐binding site, whereas in the alternate interface it is sterically excluded from the site by Phe187 33.

Another residue found at the protomer interface in yeast RAD51 is Arg357, which forms an ion pair with Glu182 of the neighbouring protomer. Both residues are strictly conserved across all the recombinase families 39. Mutagenesis work on both His352 and Arg357 in *S. cerevisiae* RAD51 has indicated that while the residues are not essential for catalysis, they likely have a role as transacting sensors of bound ATP 39, 40. As with other AAA+ superfamily members, it is possible that the ATPase centre of RAD51 and RadA acts as a conformational switch, taking an active conformation in the presence of ATP, and switching to an inactive conformation following ATP hydrolysis to ADP 37, 39, 41, 42.

The role of ATPase activity in homologous recombination is somewhat ambiguous 43, 44, 45, 46. Assays testing recombination function *in vitro* have shown that nucleotide binding, but not hydrolysis, is required for the formation of stable RAD51‐ssDNA nucleoprotein filaments 43, 44, 45, 47, 48. However, while ATP hydrolysis is not required for presynaptic filament and D‐loop formation, it is required for the completion of homologous recombination‐mediated repair *in vivo* 49. One explanation for this is that RAD51 turnover on ssDNA requires ATP hydrolysis 10, 44, 50, 51. Furthermore, D‐loop formation is enhanced in the presence of a nonhydrolysable ATP analogue rather than ATP 44. Together, these observations suggest that during recombination, ATP hydrolysis may be actively suppressed in order to maintain an active extended nucleoprotein filament. Once synapsis has been achieved, ATP hydrolysis may then be required for RAD51 turnover, thus allowing other recombination factors to access the postsynaptic stage of recombination 10, 44, 50, 51.

Here, we demonstrate that a monomerised form of human RAD51 (RAD51–BRC4 fusion) and a truncated monomeric form of *P. furiosus* RadA (RadA‐ct) retain their ability to bind ATP and other nucleotides in the low micromolar range, and we present the crystal structures of RadA‐ct bound to ATP, ADP, AMPPNP and GTP. These structures reveal that while phosphate groups are tightly bound, RadA presents only a relatively shallow and poorly defined binding surface for the nitrogenous base moieties of nucleotide ligands. In comparison with previous structures of RadA in filament form, and together with biophysical data that we present here, these findings shed light on how individual RadA protomers contribute to ligand affinity and specificity.

## Results

### C‐terminal ATPase domain of *P. furiosus* RadA is monomeric

In order to study the binding of ATP and other nucleotides to nonoligomeric RadA, we engineered the protein by removing the N‐terminal domain and the linker that contains the FxxA oligomerisation sequence (Fig. 1A). To facilitate crystallisation, the unstructured L2 DNA‐binding loop was also removed, creating a construct that we denote RadA‐ct. The resulting protein, unlike the corresponding ATPase domain of human RAD51, was solubly expressed in *E. coli* (Fig. 1B), showed high thermal stability and could be purified to homogeneity. Multi‐angle laser light scattering and nondissociative mass spectrometry showed that the protein was monomeric in solution (Fig. 1).

### Monomeric RadA binds nucleotides

Interactions of RadA‐ct with ATP, ADP, AMP, AMPPNP and GTP were assessed qualitatively using a fluorescence‐based thermal shift assay 52. Full length and RadA‐ct proved to be extremely thermostable and failed to denature at a temperature of 95 °C. A RadA mutant (HumRadA2) that was changed in the FxxA epitope‐binding site to resemble human RAD51 (mutations I169M, Y201A, V202Y, E219S, D220A and K221M) was found to have reduced thermal stability with a *T*_m_ of 81 °C. The crystal structure of this mutant showed no structural changes in the ATP‐binding site with respect to the wild‐type, full‐length protein (data not shown) and was used in subsequent thermal shift assays. Monomeric form of RAD51 in which BRC repeat 4 from BRCA2 is covalently linked to the N terminus of the ATPase domain of human RAD51 has been described before 53. The *T*_m_ of HumRadA2 and RAD51‐BRC4 in the absence and presence of ATP and other nucleotides were measured (Table 1). Binding of all nucleotide ligands was dependent on Mg^2+^. The presence of ATP or ADP increased the *T*_m_ of HumRadA2 and RAD51–BRC4 significantly, up to 10 °C. In contrast, AMP did not change the *T*_m_ of either protein and nonhydrolysable ATP analogue AMPPNP increased the *T*_m_ of HumRadA2 and RAD51–BRC4 only half as much as ATP. Thus, substitution of the hydrogen bond‐accepting oxygen between the β‐ and γ‐phosphate with a hydrogen bond donating nitrogen in AMPPNP is destabilising the complex. This is likely due to the loss of the hydrogen bond between the backbone amide of Gly‐141 and the phosphate oxygen in ATP, as evident in the crystal structure reported here.

**Table 1:** Results of the fluorescence‐based thermal shift and isothermal titration calorimetric analyses of nucleotide binding to RAD51–BRC4 and monomeric HumRadA2. The difference in the melting temperatures between the apo protein and the ligand‐stabilised proteins is denoted as Δ*T*_m_

	RAD51–BRC4		HumRadA2	
	Δ*T*_m_/°C	*K*_D_/μm	Δ*T*_m_/°C	*K*_D_/μm
ATP	9.0	1.0	10.0	1.0
ADP	7.0	1.0	7.0	0.54
AMPPNP	4.0	12.0	4.5	20.0
AMP	0.0	n/a	0.0	n/a
GTP	9.5	5.0	8.0	0.64
GDP	8.0	2.5	7.5	0.21

**Figure 2:**
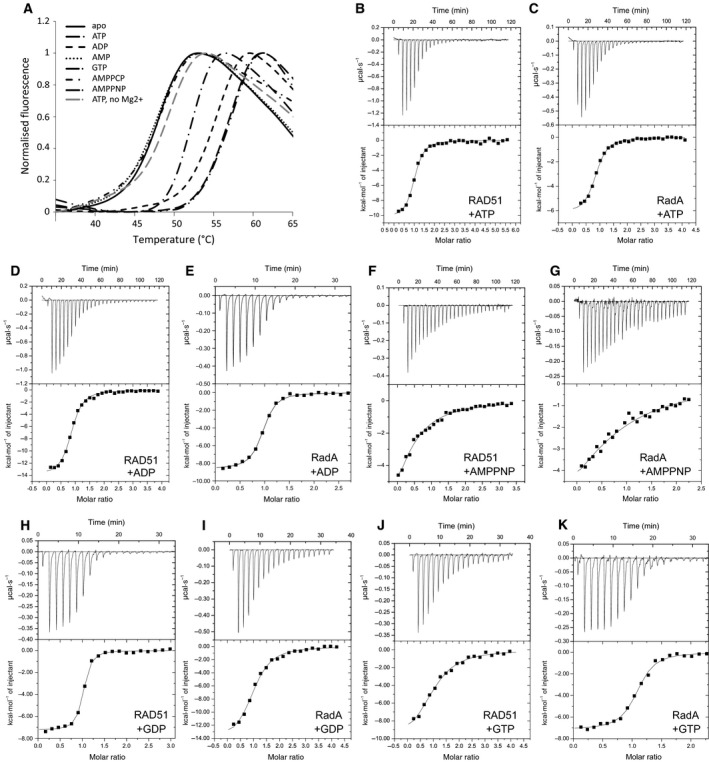
Thermal shift analysis and ITC data on nucleotide binding to RadA. (A) Normalised fluorescence traces from thermal shift analysis of RAD51–BRC4 in the presence of various nucleotides as indicated in the figure. As a control for specificity, an experiment in the presence of ATP but without Mg^2+^ is shown with dashed grey line. (B–K) ITC titrations of various nucleotides against RAD51–BRC4 (RAD51) and HumRadA2 (RadA), as indicated in each panel. Baseline‐corrected heats of binding are shown in the top panels as function of time and integrated heat (squares) for each injection and fitting to one‐to‐one binding model are shown in the bottom panels. All data from thermal shift analyses and ITC titrations are summarised in Table 1.

In order to compare the nucleotide binding more quantitatively, we determined nucleotide‐binding affinities of RAD51–BRC4 and HumRadA2 by isothermal titration calorimetry (ITC) (Fig. 2). The affinities of HumRadA2 and RAD51–BRC4 for ATP were measured at approximately 1 μm dissociation constant (*K*_D_), while affinities for AMPPNP were lower, at 20 μm and 12 μm respectively. HumRadA2 was shown to have an affinity of 0.54 μm for ADP, approximately twice as potent as for ATP. Affinities from ITC measurements mirror the results of the thermal shift results yielding similar relative order of potencies.

There is no evidence to suggest that GTP is a natural substrate for RadA. Nevertheless, we find that nonoligomeric HumRadA2 and RAD51–BRC4 can bind both GTP and GDP, and in the case of HumRadA2 with higher affinity for the adenine‐containing nucleotides with a *K*_D_ of 0.64 μm for GTP and 0.21 μm for GDP.

To analyse further the interaction with ATP, a mutant form of HumRadA2 was created in which ATP hydrolysis is blocked with a K144A mutation. Lys144 is located within the P‐loop, a universal feature of ATP‐binding proteins, binding to the triphosphate moiety of ATP 54. Mutation of this residue to alanine has previously been shown to abrogate ATP hydrolysis 55. In line with these data, binding of ATP to K144A mutant of monomeric RadA‐ct was also completely abolished, with only heats of dilution detected in ITC titration (data not shown).

### X‐ray crystal structures of nucleotide‐bound monomeric RadA

In order to determine the molecular basis of nucleotide binding by RadA, we set out to solve the X‐ray crystal structures of monomeric RadA‐ct bound to nucleotides. Firstly, we crystallised and solved the structure of RadA‐ct in the absence of nucleotides; this revealed the presence of a phosphate ion within the ATP‐binding site. We used these crystals to exchange the bound phosphates with various nucleotides by soaking the crystals in a stepwise fashion in mother liquor containing increasing concentrations of the nucleotide ligand and decreasing phosphate concentration. Using this procedure, the phosphate ion was successfully displaced from the ATP‐binding site by nucleotide ligands, thus enabling us to solve the structures of RadA‐ct bound to ATP, ADP, AMPPNP and GTP. Data collection and refinement statistics are shown in Table 2. GTP complex retained the same orthorhombic space group as the phosphate‐bound structure, whereas ATP, ADP and AMPPNP structures showed reduced symmetry and contained two molecules of RadA‐ct in the asymmetric unit; the structures of the two RadA molecules were almost identical (0.34 Å rmsd) and in nearly identical environment in the crystals, and the following discussion will focus on molecule A.

**Table 2:** Statistics of X‐ray crystallographic data collection and structure refinement

	RadA‐ct + phosphate	RadA‐ct + ATP	RadA‐ct + ADP	RadA‐ct + AMPPNP	RadA‐ct + GTP
	PDB: 4A6P	PDB: 4A6X	PDB: 4UQO	PDB: 4D6P	PDB: 4B2P
Data collection and processing					
Wavelength (Å)	0.98	0.973	0.973	0.979	0.954
Space group	P2_1_2_1_2_1_	P2_1_	P2_1_	P2_1_	P2_1_2_1_2_1_
Data collection temperature (K)	100	100	100	100	100
a, b, c (Å)	60.92, 40.18, 87.22	40.32, 87.35, 61.88	40.03, 87.13, 62.32	40.45, 87.42, 62.22	40.32, 62.02, 87.18
α, β, γ (°)	90.0, 90.0, 90.0	90.0, 91.2, 90.0	90.0, 90.5, 90.0	90.0, 91.5, 90.0	90.0, 90.0, 90.0
Resolution range (high resolution bin) (Å)	43.61–1.50 (1.58–1.50)	43.68–1.55 (1.63–1.55)	43.40–1.88 (1.99–1.88)	40.44–1.48 (1.57–1.47)	36.59–1.60 (1.70–1.60)
*R*_*sym*_	0.070 (0.550)	0.089 (0.644)	0.103 (0.504)	0.070 (0.515)	0.097 (0.821)
Completeness (%)	92.4 (87.7)	95.7 (93.7)	99.1 (96.6)	93.9 (93.8)	99.7 (98.3)
Number of unique reflections	32 321	59 655	34 773	68 062	29 390
Redundancy	4.8 (4.5)	3.7 (3.7)	3.5 (3.5)	3.7 (3.6)	7.1 (7.0)
<*I*/σ(*I*)>	17.4 (3.6)	11.5 (1.9)	10.4 (2.6)	12.6 (2.8)	17.8 (3.1)
Wilson B‐factor (Å^2^)	8.0	10.4	24.3	19.5	23.1
Refinement					
Resolution range (Å)	26.59–1.50	43.68–1.58	43.40–1.88	40.443–1.48	36.59–1.60
Number of reflections: work/test set	31 691/1623	59 636/2899	34 773/1736	64 084/3372	27 899/1490
*R*_cryst_ */R*_free_	0.184/0.214	0.196/0.235	0.171/0.231	0.193/0.239	0.179/0.220
Number of protein atoms	1866	3683	3568	3585	1701
Number of other atoms	432	627	496	674	117
Average B factors protein/other atoms (Å^2^)	11.8/28.2	13.7/27.9	21.6/33.2	15.3/27.6	15.1/29.0
Ramachandran analysis					
Favoured/allowed/outliers	210/1/0	433/2/0	425/9/0	437/8/2	210/0/0
Model geometry					
RMSD bonds (Å)	0.006	0.006	0.017	0.008	0.026
RMSD angles (°)	1.026	1.115	1.792	1.213	2.153

The ATP‐binding sites are very similar in all the nucleotide‐bound and non‐nucleotide–bound RadA structures, in the most part with only minor differences in the positioning of the side chains. One notable exception to this is the positioning of the Phe140 side chain which in the ADP and non‐nucleotide–bound structures points towards the nucleotide. In the ATP and GTP complexes, this side chain has an alternative rotamer pointing away from the nucleotide.

**Figure 3:**
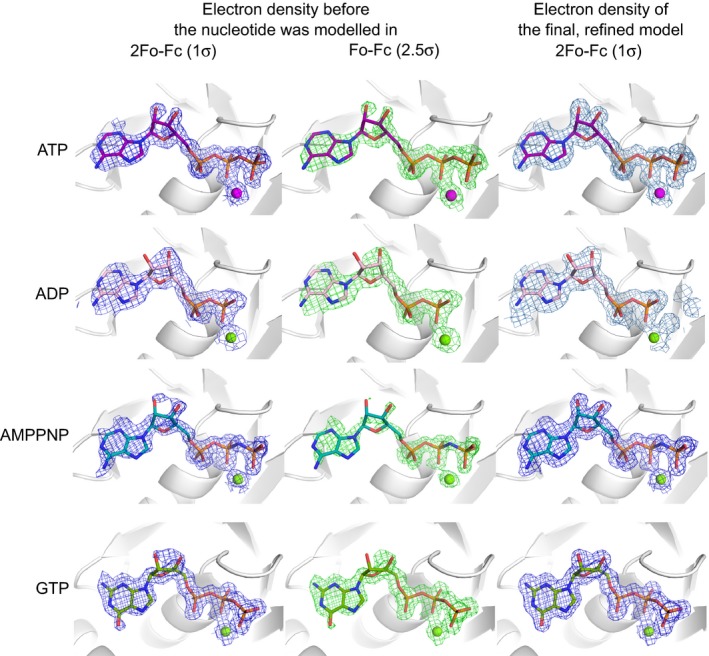
Electron density maps of the bound nucleotides. Weighted 2FoFc and FoFc electron density maps shown in blue (countered at 1σ) and green (2.5 σ), respectively in the left and middle columns for each of the nucleotides *before* the nucleotide was modelled into the structure. The right column shows the final weighted 2FoFc maps for each of the modelled nucleotides and their associated Mg^2+^ ions.

**Figure 4:**
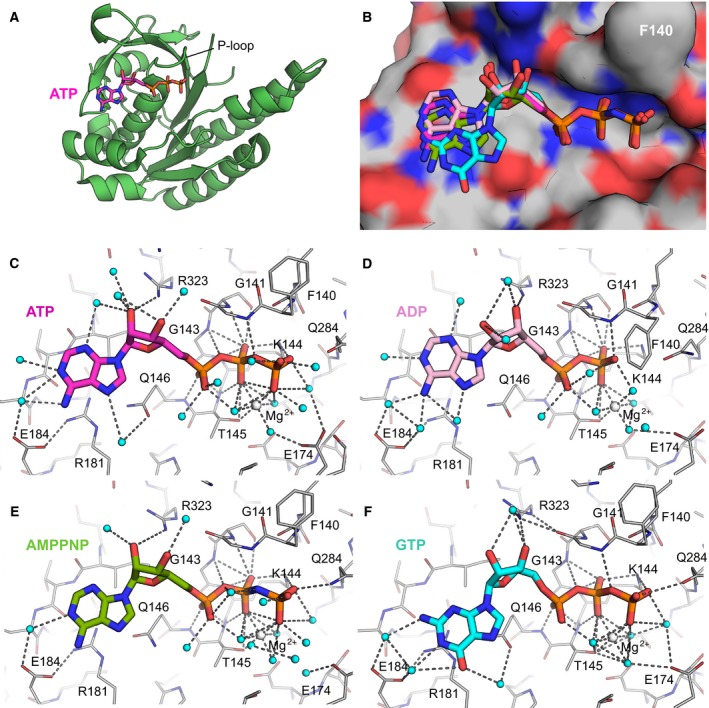
Crystal structures of nucleotide‐bound monomeric RadA. (A) Overview of RadA‐ct bound to ATP. (B) Overlay of all nucleotides in superimposed RadA‐ct complexes. Nucleotides are shown in stick representation: ATP (purple), ADP (pink), AMPPNP (green), GTP (cyan). Protein shown as surface molecule is that complexed with ATP, with F140 labelled to aid with orienting it with respect to the remaining figures. (C–F) Hydrogen bonding interactions between RadA‐ct and ATP (C), ADP (D), AMPPNP (E) and GTP (F). Water molecules are shown as cyan spheres and Mg^2+^ ions as white spheres. Colouring of nucleotides as in (B).

Electron density was clearly visible for all nucleotides in the electron density maps, before these ligands were included in the models (Fig. 3). The phosphate ion present in the native RadA‐ct structure is located in the position occupied by the β‐phosphate group of nucleotide‐bound structures. All nucleotides bind with very similar conformations, with the highest variability occurring in the nucleoside region. This likely reflects the fact that most nucleotide‐binding contacts of RadA‐ct are between the P‐loop of RadA and the phosphate groups of the nucleotide, whereas the nucleoside part is located on relatively wide and flat surface on the protein (Fig. 4A,B).

The phosphate regions of all nucleotide ligands make similar contacts with RadA, albeit with several notable differences. The α‐phosphates form interactions with the main chain amides of Gln146 and Thr145, and the β‐phosphates form comparable interactions with the side chain of Lys144 and both the side chain and backbone of Thr145 (Fig. 4C–F). Residues equivalent to Lys144 and Thr145 have been shown to be important for enzymatic function of bacterial RecA 56. The β‐phosphates of ATP and ADP form a hydrogen bond with the main chain amide of Gly141, an interaction that is presumably weakened by the presence of the nitrogen of AMPPNP in this position. Gly143 interacts with the oxygen bridging between β‐ and α‐phosphates in all complexes. The terminal γ‐phosphates of ATP and AMPPNP both form bonds with the side chains of Gln284 and Lys144; the equivalent residue of Gln284 in bacterial RecA (Gln194) has previously been shown to be required for the ATPase activity 57, 58.

A magnesium ion is observed in all nucleotide‐bound structures. It is positioned in the same location in ATP and AMPPNP structures, co‐ordinated by the β‐ and γ‐phosphate groups and the hydroxyl group of Thr‐145. In the ADP structure, the Mg^2+^ ion is shifted slightly towards the β‐phosphate group; it is also co‐ordinated by the β‐phosphate and the hydroxyl group of Thr‐145, in addition to four water molecules. The residue Glu174 likely acts as a counterpart to Glu96 of *E. coli* RecA, which is thought to activate a water molecule for hydrolysis of ATP 27. In our structures, Glu174 forms an indirect interaction with the Mg^2+^ ion via a bridging water molecule (Fig. 4c). As magnesium was not present in the crystallisation conditions of RadA, the phosphate‐bound form also lacks magnesium, but this appears to have little impact on the binding of the phosphate.

There are fewer contacts between the nucleoside moiety and the protein; this part of the binding pocket is flat, with the nucleoside stacked against Arg181. The ribose groups of all the adenine‐containing nucleotides form hydrogen bonds with the side chain of Arg323. No hydrogen bond interactions are formed between the bases of any of the nucleotides and the protein.

Comparison of the ATP‐binding site of our monomeric *P. furiosus* RadA‐ct structures with that of nucleotide‐bound filamentous RadA structures 34, 35, 37, 59 reveals only minor differences, demonstrating that nucleotide binding of the monomeric protein is not merely an artefact of crystallisation. We do observe a difference in the conformation of the backbone of the loop region between residues 342 and 346. In contrast to our monomeric RadA structures, in nucleotide‐bound filament structures, this loop is shifted towards the nucleoside, with a corresponding shift in the adenine moiety.

## Discussion

We have determined the crystal structures of a truncated monomeric form of *P. furiosus* RadA (RadA‐ct) bound to ATP, ADP, AMPPNP and GTP. These structures reveal that the RadA monomer is capable of binding nucleotides in the absence of an adjacent protomer. Using ITC and thermal shift methods, we have obtained quantitative data for the interaction with different nucleotides for both *P. furiosus* RadA and human RAD51.

Both monomeric RadA‐ct and RAD51 are able to bind ATP, ADP, AMPPNP, GTP and GDP in an Mg^2+^‐dependent manner. In comparison, AMP is not able to bind to either of the proteins indicating that both the β‐ and γ‐phosphates are essential for this interaction. ADP and GDP bind to RadA with approximately twice the affinity of their triphosphate equivalents. This could possibly be explained by the different conformation of the side chain of Phe140 between the di‐ and triphosphorylated nucleotide complexes, with the additional negative charge of the γ‐phosphate repelling the aromatic ring. AMPPNP does bind to RadA‐ct and RAD51, but with reduced affinity in comparison with ATP. This is likely to be caused by the inability of the nitrogen between the β‐ and γ‐phosphates to form a hydrogen bond with the backbone amide of Gly141 as observed in the complex with ATP (Fig. 4c).

It is inappropriate to compare either the magnitude or relative order of binding constants between RadA‐ct and RAD51‐BRC4 because neither set of measurements were made at physiological temperature. Even a simplistic extrapolation of the free energy of binding for *Pf*RadA to its optimal growth temperature (373 K) translates to binding constants in the range 1–10 μm 60. However, both the magnitude and relative order of binding free energies would be significantly affected by a nonequivalent and nonzero heat capacities (Δ*C*_p_) of ligand binding in the different interacting systems (RadA versus RAD51, ATP versus ADP, etc.).

The positioning of the Phe140 side chain differs between the structures. In the ADP and non‐nucleotide–bound structures, the side chain points towards the nucleotide. In the ATP and GTP structures, it has an alternative rotamer pointing away from the nucleotide. The equivalent Phe187 residue in *S. cerevisiae* RAD51 (*Sc*RAD51) was shown to take a different conformation in the two alternative protomer–protomer interfaces observed in the crystal structure of the filament form 33. Similar change in the side chain position of the equivalent Phe is also observed in the *Methanococcus voltae* RadA (*Mv*RadA) structures between the AMPPNP‐ and ADP‐bound forms 37.

**Figure 5:**
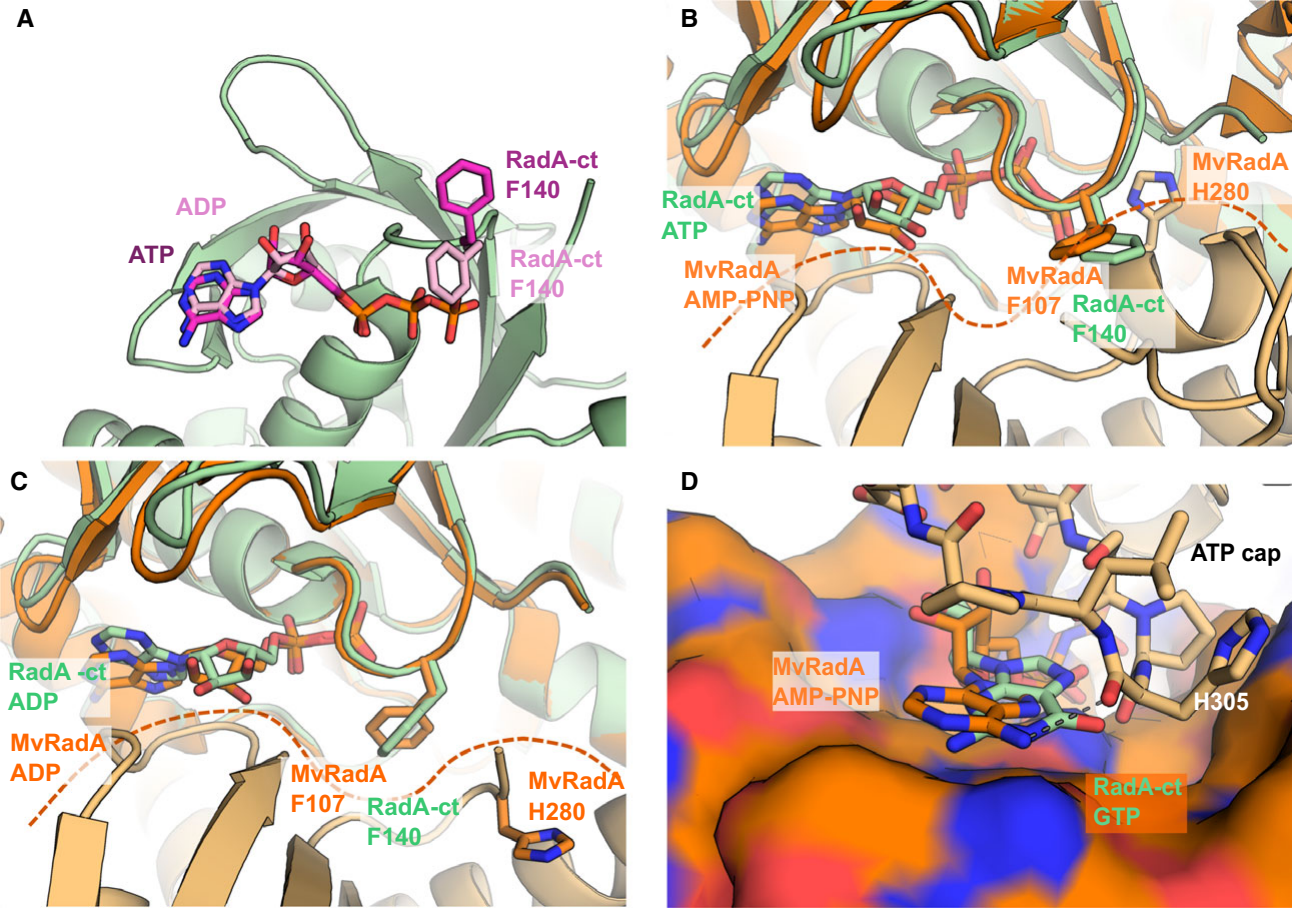
Comparison of monomeric RadA‐ct nucleotide complexes with filament structures of *Methanococcus voltae* RadA. (A) Superimposed nucleotide‐bound RadA‐ct structures with ATP and ADP. Nucleotides shown in stick representation – ATP (purple), ADP (pink). The F140 residue is shown in stick representation to illustrate the differing conformation between the ATP‐bound (purple) and ADP‐bound structures (pink). (B) Superimposed ATP‐bound monomeric RadA‐ct (light green) and AMPPNP bound to *Methanococcus voltae* RadA (*Mv*RadA) oligomer with the adjacent RadA molecules coloured in darker and lighter orange (PDB: 2FPM). *Mv*RadA residues and AMPPNP are shown in orange, *Pf*RadA residues and ligand in light green. Superpositioning was done between RadA‐ct and the *Mv*RadA protomer shown on the top, and the interface between the two *Mv*RadA molecules is indicated with a dashed line. (C) Superimposed ADP‐bound RadA‐ct and ADP‐bound *Mv*RadA molecules (PDB: 2FPK), coloured as in panel (B). (D) Superimposed structures of GTP‐bound RadA‐ct and AMPPNP‐bound *Mv*RadA oligomer (PDB: 2FPK). One of the protomers in the *Mv*RadA oligomer is shown as molecular surface and the ATP loop of the adjacent protomer is shown as sticks. Hydrogen bond between main chain oxygen and the adenine N6‐exoamino group of AMPPNP is shown as a dashed line.

Comparison of the Phe140 position between our ATP and ADP structures with the equivalent Phe107 in the AMPPNP and ADP *Mv*RadA structures is shown in Fig. 5 (A–C). The Phe has the same rotameric form in both ADP structures pointing in towards the nucleotide‐binding site, whereas in both the ATP and AMPPNP structures, the side chain is pointing away from the nucleotide‐binding site. As with the *Sc*RAD51 structure, the positioning of Phe107 in the *Mv*RadA ADP structure effectively occludes His280 (equivalent to His352 in *Sc*RAD51) of the neighbouring protomer from the nucleotide‐binding site. This observation adds weight to the idea that His352 has a regulatory role as a transacting sensor of ATP presence possibly mediated by the positioning of the Phe.

An overlay of our ATP structure with an ATP‐bound filament form of *Mv*RadA shows that the ATP cap region (residues 301–308 in *Mv*RadA, 328–335 in *Pf*RadA) of the second protomer would make extensive contacts with the adenine and ribose moieties of the ATP (Fig. 5D) 35. Wu *et al*. 35 has suggested that the preference of *Mv*RadA for ATP binding may be due to the interactions of the adenine N6 atom with the side chain of Gln161, and the carbonyl oxygen of His305 from the ATP cap region. Although there is no evidence to suggest that GTP is a natural substrate for RadA or RAD51, it was found that the guanine‐containing nucleotides bind to the monomeric form of RadA and RAD51 with a somewhat higher affinity than the adenine‐containing equivalents. It is not clear from our crystal structures why this is the case, and we can only speculate that the guanine base makes additional hydrophobic interactions with the protein. GTP does not have a hydrogen bond donor at the equivalent position of the N6 atom of ATP and so would be unable to form these interactions.

In our structures, none of the nucleotides interacts with Glu184, the equivalent residue to Gln161 in *Mv*RadA. Comparison of nucleotide‐bound structures of RadA filaments with our structures shows a shift of the loop region between residues 342 and 346 towards the position of the bound nucleotide, causing a corresponding shift in the position of the adenine. It is possible that the conformation of this loop region changes upon binding of the second protomer. This could cause a shift in the position of the nucleotide allowing it to interact with Glu184 and with His332 in the ATP cap of the neighbouring protomer and would have the effect of fixing the position of the adenine. The selectivity for ATP over GTP appears therefore to be mediated by the binding of the second protomer, which forms specific interactions with ATP but not with GTP.

The fact that RadA is able to bind nucleotides in the absence of an adjacent protomer suggests that RadA exists in the cell in a nucleotide‐loaded state prior to filament formation. Intracellular ATP concentration is estimated to be in low millimolar range, and with a dissociation constant of 1 μm, RadA would be fully saturated with the ligand. Similarly, eukaryotic RAD51 would exist predominantly in nucleotide‐bound form, also when complexed with BRC repeats. Given the interfacial location of ATP in the RAD51 filament, it is likely that bound nucleotide will facilitate self‐association of RadA and RAD51 and the assembly of an active form of the filament. Although the nucleotide‐binding and FxxA epitope‐binding sites are located on the opposite sides of the ATPase domain, they are separated by only 20 Å through the structure. It is possible that nucleotide binding could have an allosteric and/or co‐operative effect on the binding to FxxA sequences and play an additional role in regulating the oligomeric structures of these recombinases, but further work is needed to elucidate these effects and their significance to the biological function of RAD51 and RadA.

## Experimental procedures

### Protein expression and purification

The C‐terminal ATPase domain of *P. furiosus* RadA, starting from residue 108 and lacking residues 288–301 in the so‐called L2 loop (RadA‐ct), was expressed from T7‐based expression plasmid pBAT4 61 in BL21(DE3) *Escherichia coli* strain carrying pUBS520 plasmid for rare Arg tRNA 62. Bacteria were grown at 37 °C until OD_600_ reached 0.8–1.0 and expression was induced with 400 μm of IPTG. After 3 h, cells were centrifuged and cell pellets stored at −20 °C. Thawed cells were resuspended in 20 mm MES pH 6.0 buffer, disrupted using Emulsiflex C5 and heat‐treated for 20 min at 70ᵒC in order to denature the *E. coli* host proteins. Denatured protein was removed by centrifugation and clarified lysate, containing thermostable RadA, was loaded onto a 5 mL HiTrap SP‐HP cation‐exchange column (GE Healthcare Life Sciences, Little Chalfont, UK) pre‐equilibrated with 20 mm MES pH 6.0, 0.5 mm EDTA. Protein was eluted using a gradient of NaCl. Peak fractions were concentrated and loaded onto a Superdex 75 16/60 HiLoad size‐exclusion column (GE Healthcare) and eluted using 20 mm MES pH 6.0, 100 mm NaCl, 0.5 mm EDTA. The human BRC4–RAD51 fusion protein was purified according to the previously described procedure 53.

### Fluorescence‐based thermal shift analysis

The thermal shift denaturation assay was performed on an iCycler iQ Real‐Time Detection System (Bio‐Rad Laboratories Ltd, Hemel Hempstead, UK) in 96‐well plates and iCycler iQ PCR plates sealed with optically clear lids. The fluorescent dye Sypro Orange was used to report protein unfolding. For the RadA protein, each well contained the protein at a final concentration of 13.2 μm, 2.5× Sypro Orange, 20 mm Tris, pH 7.5, 40 mm NaCl and 5 mm MgCl_2_, in a total volume of 100 μL. Similar conditions with the human RAD51 protein were used; except the protein was used at 10.5 μm and the buffer contained 100 mm NaCl. Unless otherwise stated, nucleotides were used at 1 mm. The plates were heated at a rate of 0.5 °C·min^−1^ from 25 to 90 °C, monitoring the fluorescence continuously with excitation at 490 nm and emission at 530 nm. To determine the thermal denaturation temperature, *T*_m_, for each well, the minimum of the negative derivative of the thermal melting curve was determined.

### Isothermal titration calorimetry

ITC experiments were performed at 25 °C on a VP‐ITC instrument from Microcal LLC (Northampton, MA, USA). Protein was buffer exchanged into 50 mm Tris, pH 7.5, 100 mm NaCl and 5 mm MgCl_2_. In a typical experiment, 30 μm enzyme was loaded in the sample cell and a total of twenty‐eight 10 μL injections of 8 s in duration were made at 4‐min intervals from a syringe rotating at 300 rpm loaded with 400 μm nucleotide. In all titrations, an initial injection of 2 μL ligand were made and discarded during data analysis. Control titrations of ligand to buffer were performed and subtracted from the ligand to protein titrations. The thermodynamic parameters were obtained by fitting the data to a single site‐binding model using the ITC analysis package in origin 5.0 (OriginLab, Northampton, MA, USA).

### Crystallisation, X‐ray diffraction and structure determination

Protein was concentrated to 10–15 mg·mL^−1^ for crystallisation. The RadA ATPase domain (RadA‐ct) was crystallised by the hanging‐drop vapour diffusion method using 60 mm Na_2_HPO_4_, 15% PEG1000 as precipitant. Crystallisation was performed at 20 °C. In order to determine the structure of ligand‐bound RadA, crystals were soaked with different nucleotides. The mother liquor contained 25% (v/v) glycerol, 15% PEG1000, 10 mm MgCl_2_ throughout and Na_2_HPO_4_ concentration was decreased from 50 to 10 mm, while nucleotide concentration was increased from 2 to 10 mm in five steps. The final soaking solution had the composition: 25% glycerol, 15% PEG1000, 10 mm MgCl_2_, 10 mm Na_2_HPO_4_, 10 mm nucleotide. Crystals were soaked in each of the soaking solution for 10 min and flash‐cooled in liquid nitrogen after the final soak.

X‐ray diffraction data were collected on beamline I04 of the Diamond Light Source from apo‐RadA‐ct and ATP‐ and ADP‐bound RadA‐ct crystals and at beamline ID23‐1 of the European Synchrotron Radiation Facility, Grenoble, France from RadA‐ct‐GTP crystals and at beamline X06DA/PXIII, at the Swiss Light Source from RadA‐ct AMPPNP crystals. Images were indexed, scaled and integrated using the program XDS 63. The data‐processing statistics are shown in Table 2.

The phosphate‐bound RadA‐ct crystallised in an orthorhombic space group with one molecule in the asymmetric unit. With the exception of the GTP complex, soaking of the nucleotides into the crystal reduced the symmetry of the crystals and these structures were found to be in a monoclinic P2_1_ space group with two molecules in the asymmetric unit. The crystal structure of nucleotide‐free RadA was solved by molecular replacement using the program AMoRe 64 with the co‐ordinates of the *P. furiosus* RadA as a probe (PDB: 1PZN, chain A). The nucleotide‐bound structures were solved using molecular replacement with the unliganded RadA as a model. One round of refinement was performed using REFMAC5 65 and the model was built using Coot 66. The electron densities for each of the nucleotide ligands and the co‐ordinated Mg^2+^ ion were clear and allowed their unambiguous placement in the model (Fig. 3). No electron density was evident for the loops Gln286 to His307 for the ATP‐ and ADP‐bound structures and between Gln286 to His303 for the AMPPNP structure. Water molecules were introduced and several more rounds of refinement were performed using Phenix 67. Analysis of the diffraction data suggest that some of data sets from monoclinic crystals forms are twinned, but refinement taking this into account showed no significant improvement in the models or statistics. The stereochemistry of the models was validated using Coot 66. As the monoclinic crystals are closely related to the orthorhombic form, all proteins in these structures are in nearly identical environment in the crystals and no significant differences were found between the two molecules in the monoclinic structure. All the figures and analyses have been done using protein chain A of each structure.

Co‐ordinates were deposited in the Protein Databank under the following accession numbers: PDB: 4A6P (apo–RadA‐ct), PDB: 4A6X (ATP‐bound RadA‐ct), PDB: 4UQO (ADP‐bound RadA‐ct), PDB: 4D6P (AMPPNP‐bound RadA‐ct) and PDB: 4B2P (GTP‐bound RadA‐ct).

## Author contributions

MM, DS and ME performed all the experiments. MM, DS, ME, CA, TLB and MH conceived the study, designed the experiments and interpreted the results. All authors contributed to the writing of the manuscript.
